# Strategies to eradicate HIV from infected patients: elimination of latent provirus reservoirs

**DOI:** 10.1007/s00018-019-03156-8

**Published:** 2019-05-25

**Authors:** Ivan Sadowski, Farhad B. Hashemi

**Affiliations:** 1 Department of Biochemistry and Molecular Biology, University of British Columbia, 2350 Health Sciences Mall, Vancouver, BC V6T 1Z3 Canada; 2 Department of Microbiology, School of Medicine, Tehran University of Medical Sciences, Tehran, Iran

**Keywords:** AIDS, HIV, Cure, Anti-retroviral therapy, Virus latency, Latency-reversing agents, Shock and kill, Transcriptional regulation, Lock and block, Genome editing, Immune modulation

## Abstract

35 years since identification of HIV as the causative agent of AIDS, and 35 million deaths associated with this disease, significant effort is now directed towards the development of potential cures. Current anti-retroviral (ART) therapies for HIV/AIDS can suppress virus replication to undetectable levels, and infected individuals can live symptom free so long as treatment is maintained. However, removal of therapy allows rapid re-emergence of virus from a highly stable reservoir of latently infected cells that exist as a barrier to elimination of the infection with current ART. Prospects of a cure for HIV infection are significantly encouraged by two serendipitous cases where individuals have entered remission following stem cell transplantation from compatible HIV-resistant donors. However, development of a routine cure that could become available to millions of infected individuals will require a means of specifically purging cells harboring latent HIV, preventing replication of latent provirus, or destruction of provirus genomes by gene editing. Elimination of latently infected cells will require a means of exposing this population, which may involve identification of a natural specific biomarker or therapeutic intervention to force their exposure by reactivation of virus expression. Accordingly, the proposed “Shock and Kill” strategy involves treatment with latency-reversing agents (LRA) to induce HIV provirus expression thus exposing these cells to killing by cellular immunity or apoptosis. Current efforts to enable this strategy are directed at developing improved combinations of LRA to produce broad and robust induction of HIV provirus and enhancing the elimination of cells where replication has been reactivated by targeted immune modulation. Alternative strategies may involve preventing re-emergence virus from latently infected cells by “Lock and Block” intervention, where transcription of provirus is inhibited to prevent virus spread or disruption of the HIV provirus genome by genome editing.

## Introduction

Over 37 million people are currently living with Human Immunodeficiency virus (HIV), and ~ 2.1 million new infections per year continues to fuel the HIV pandemic [1], for which there is no cure. HIV infection initially causes acute mild symptoms which, if left untreated, usually leads to a chronic disease characterized by gradual depletion of immune function such that infected individuals ultimately succumb to a variety of opportunistic secondary infections. Since HIV does not infect animals, elimination of this virus from the human population is scientifically plausible [2]. However, this will require concerted efforts that must address control of HIV spread within the population, as well as the elimination of persistent virus within infected individuals. Control of spread involves comprehensive global control measures to identify and treat infected people worldwide, plus strategies to prevent transmission to the uninfected population. Moreover, the elimination of persistent HIV within infected patients will require effective treatments that eradicate cells harboring latent provirus genomes. To date, noteworthy HIV eradication programs such as UN/AIDS 90-90-90, which utilize global epidemiological strategies and mathematical models, have been implemented by the World Health Organization (WHO) with the goal of controlling HIV disease among the global population, focusing on developing nations [3, 4]. Within infected individuals, despite the remarkable successes in reducing morbidity and mortality of people living with HIV, eradication of the virus is limited by persistent latently HIV-infected cellular reservoirs. This review focuses on the cellular and viral aspects of HIV latency and describes potential strategies for eliminating these latent reservoirs.

HIV is a retrovirus that represents one of the most tenacious challenges humans have encountered in recent history, but probably only the most recent confrontation with retroviruses throughout our evolution. The human genome is littered with remnants of retroviral sequences that exist as evidence of an ongoing co-existence of transposable genetic elements and their host eukaryotic organisms [5]. Consequently, in several millennia, should humankind survive, it can be expected that fragments of HIV provirus might become recognizable as stable elements of human genome. HIV/AIDS became part of public consciousness in the early 1980’s, and within 15 years it not only developed into a devastating global pandemic that meant inevitable death for most infected individuals but also exaggerated social divisions because of the predominantly affected populations. Development of combination anti-retroviral therapies (ART) in the mid 1990’s significantly improved prognosis for most infected people, who can now live relatively normally, but are destined to remain on medication for the duration of their lives [6, 7]. HIV infection in most cases no longer represents a death sentence, and individuals have now been living with the virus for 20 or more years. Unfortunately, a consequence of these effective therapies is that public awareness of HIV/AIDS has diminished, and infection rates are again increasing, particularly in developing nations of Africa and Asia, but also marginalized communities in North America and Europe, including in many instances aboriginal populations [8, 9]. An increasing awareness that current therapies are a short-term solution, and growing health problems among the aging HIV-infected population [10], has led to a discussion of potential “cures” for HIV infection [11]. Given that ~ 35 million people have died from HIV/AIDS-related disease, and currently nearly 40 million are living with HIV-infection, this represents the most significant infectious disease for which a pathogen has been identified but curative therapy has yet to be devised.

## HIV infection and the development of latent provirus reservoirs

HIV infects a variety of immune cell types bearing the CD4 and CXCR4/CCR5 co-receptors, including helper T cells, macrophages, and dendrocytes, and if untreated, microglial cells and astrocytes of the nervous system (Table 1). Latent provirus can presumably develop in all of these cells, but the mechanisms for the establishment of latency is best understood in the CD4^+^ T cell population. These memory T helper cells become programmed with specific antigens from a naïve precursor state through the engagement of the T cell receptor with antigen presenting dendritic cells. Programmed T helper cells evoke a corresponding immune response, and ultimately revert to a resting latent state upon clearance of antigen [12]. The extremely long lifespan of infected resting memory T cells represents a barrier to the elimination of the infection by current antiretroviral therapies [13]. However, since a variety of additional cell types can be infected by HIV (Table 1), it is recognized that the complete reservoir of cells harboring HIV provirus in infected patients has not been precisely determined [14, 15].

**Table 1:** HIV target cell types and tissue distribution

Cell lineage	Markers	Tissue reservoirs	Life span	References
CD4^+^ T lymphocytes	CD4, CD45, CXCR4, CCR5/CCR3	Peripheral blood, lymphatic tissue, gastrointestinal tract	1–3 years	[36, 37]
Cytotoxic CD8^+^ T lymphocytes	CD8	Peripheral blood, lymphatic tissue, gastrointestinal tract	1–3 years	[36–38]
Monocytes	CD4, CD14, CD16, CD52, CXCR4	Peripheral blood, lymphatic tissue	4–7 days	[15]
Macrophages	CD4, CD13, CD11b, FcγR	Peripheral blood, lymphatic tissue	2–24 months	[39]
Dendrocytes	CD4, CD16, CD14, CD1c, CD141	Peripheral blood	2–14 days	[14, 15]
Folicular Dendrocytes^a^	CD4, CD14, CD1c, CD141	Lymphoid tissue	2–14 days	[40]
Microglia	CD4, CD45, CD11b, P2RY12	Central nervous system	3–10 years	[41]
Astrocytes^b^	CD44, GLAST, ACSA	Central nervous system	Months	[39]
Perivascular macrophages	CD4, CD45, CD206	Central nervous system	Months	[42]
Adipose macrophages	CD4, CD206, CD14	Adipose tissue	2–24 months	[15]
Kupfer cells	CD4, CD68, CD11b	Liver	3–4 days	[39]
Epidermal Langerhans	CD4, CD1a, CD207	Skin epidermis, genital tract	Months	[39]
HSPCs	CD4, CD34, CD133	Bone marrow	Years	[43]
Epithelial cells^b^	CD146, CD326	Genital tract, mammary tissue	Years	[14]

^a^Can maintain virus on surface without becoming infected

^b^May become infected by syncitia formation

**Fig. 1:**
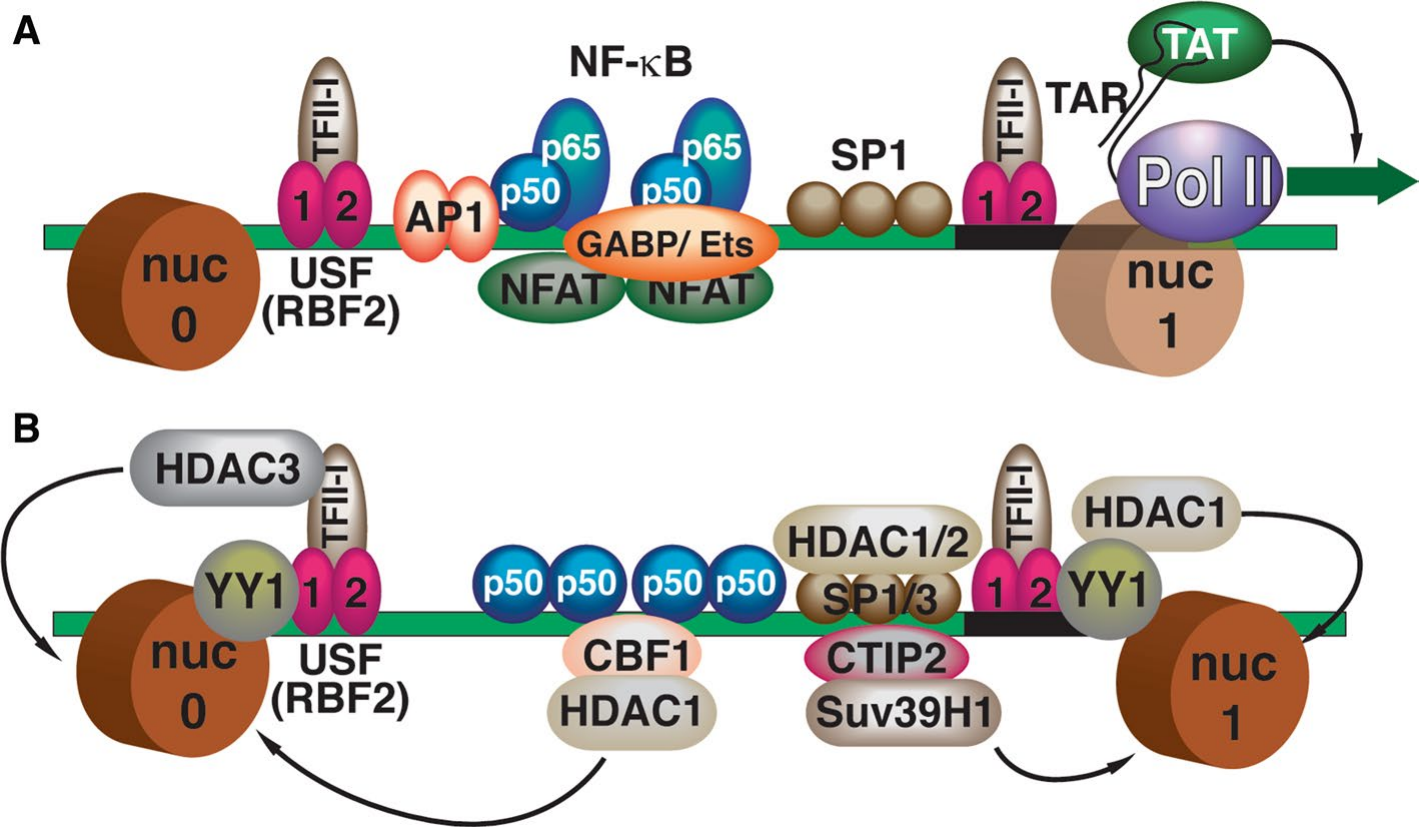
Transcription factors controlling activation and repression of the HIV-1 LTR. **a** Transcription factors mediating the activation of HIV-1 transcription. The enhancer region of the 5′ HIV-1 LTR binds multiple transcriptional activator proteins [AP1, NF-κB, SP1, NFAT, GABP/Ets, USF1/2/TFII-I (RBF-2)] that recruit general transcription factors and co-activator complexes to stimulate transcription by RNA Polymerase II (Pol II). Transcription of the HIV-1 5′ mRNA region produces the TAR (TAT-Responsive) RNA stem-loop structure that binds the viral TAT protein, which recruits elongation factor pTEFB to inhibit negative regulators of pausing, DSIF and NELF, and promote elongation by RNA Pol II. The 5′ LTR is associated with two strongly positioned nucleosomes, designated nuc-0 and nuc-1; transcriptional activation from the LTR is causes dissociation of nuc-1 near the core promoter. **b** Factors causing repression of HIV-1 transcription in unstimulated cells. In unstimulated cells, activator proteins are replaced by transcriptional repressors (NF-κB p50, CBF-1) that recruit histone deacetylase and histone methyltransferase complexes. Several LTR-bound factors are converted from activators to repressors (SP1/3 (RBF-2)/TFII-I) that recruit HDAC enzymes (HDAC1/2/3). The multifunctional factor YY1 (Yin Yang 1) is associated with the latent provirus 5′ LTR and also recruits HDACs. Several factors, including CTIP-2, recruit histone methyltransferases (Suv39H1) that promote transcriptional silencing and spreading of repressive chromatin

**Fig. 2:**
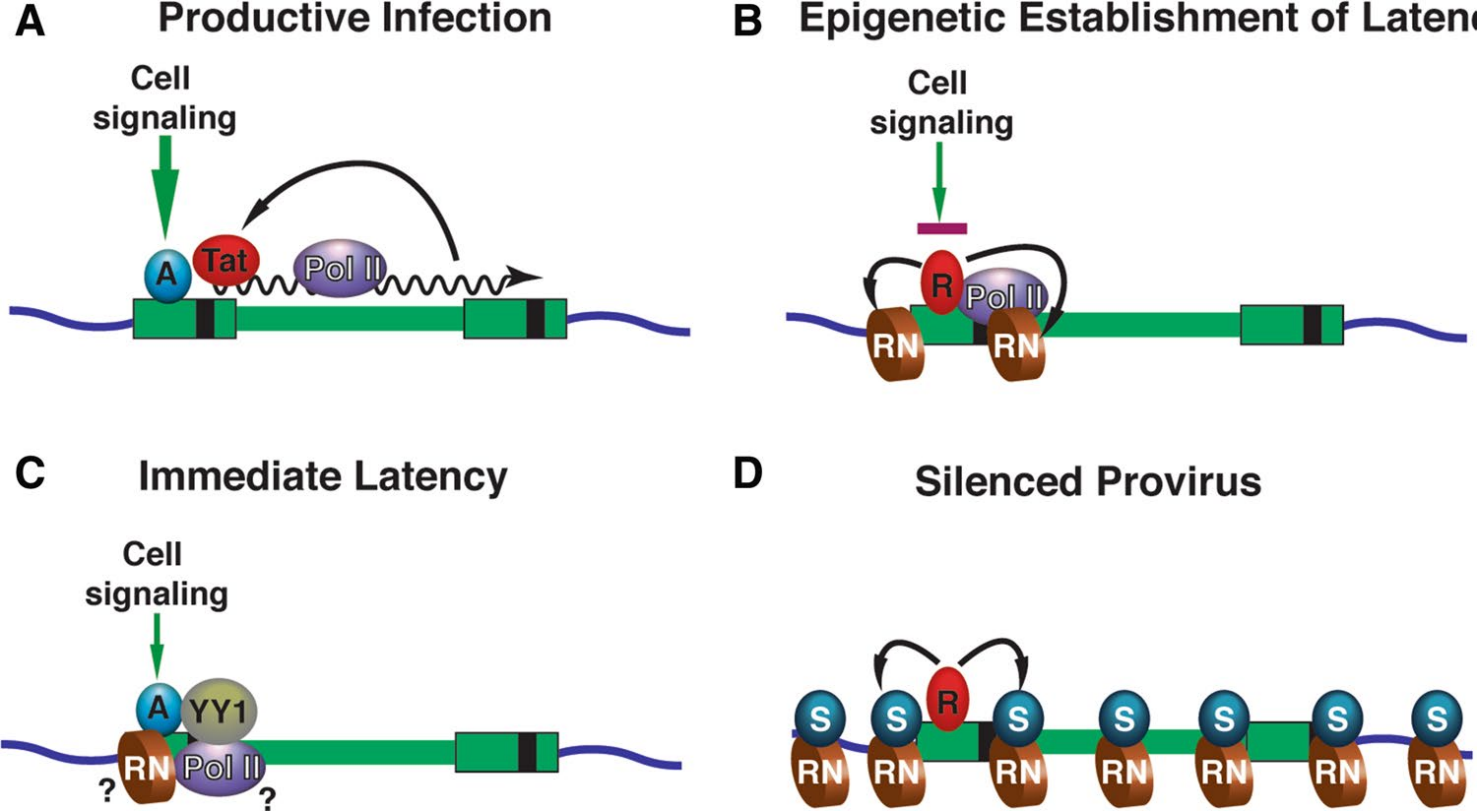
Mechanisms for the establishment of HIV-1 provirus latency. **a** Productive infection; in productively infected cells, expression from integrated HIV-1 provirus is stimulated by signal-responsive (cell signaling) transcriptional activators (A) bound to the 5′HIV-LTR enhancer. The viral transactivator Tat produces strong positive feedback activation of LTR-directed transcriptional elongation by RNA Polymerase II (Pol II) to maintain productive infection. **b** Epigenetic establishment of latency; in cells where signaling is down-regulated (T cells that revert to latency) signal-responsive transcriptional activators are replaced with repressor proteins (R) that recruit histone modifying factors which cause the formation of repressive nucleosomes (RN) through histone deacetylation and histone methylation. Repressive chromatin inhibits transcriptional initiation and elongation from the 5′ LTR promoter. **c** Immediate latency; approximately 50% of newly infected cells produce integrated HIV provirus where LTR transcription is repressed within 24 h. Immediate latency is associated with low levels of cell signaling, and requires the interaction of YY1 with the 5′ LTR, but the mechanism(s) producing this mode of latency have yet to be determined (?). **d** Silenced Provirus; repressor proteins (R) bound to the transcriptionally repressed 5′ HIV LTR recruit silencing complexes which promote spreading of silenced (S) heterochromatin onto adjacent viral and cellular chromosomal DNA

The mechanisms for the establishment of latent HIV provirus in T cells have been the subject of many recent excellent and detailed reviews [13–16]. Briefly, transcription from the HIV-1 long terminal repeat (LTR) promoter (Fig. 1) is tightly linked to signaling pathways downstream of receptors involved in immune cell activation, including those involved in response to cytokines and antigen presentation [17, 18]. The HIV-1 LTR enhancer region is inundated with binding sites for factors responsive to these signaling pathways, including AP1, TFII-I, GABP/Ets, NFAT, and NFκB (Figs. 1a, 2a), and importantly activity of these factors becomes down-regulated in un-stimulated cells or memory cells that have reverted to latency [18, 19]. Additionally, many of these *cis*-elements which are occupied by transactivators in stimulated cells, are replaced by factors that recruit repressive complexes in un-stimulated cells (Fig. 1b), including histone deacetylates and histone methyltransferases [20]. Accordingly, mechanisms mediating epigenetic silencing of HIV transcription are well-documented (Fig. 2b), and evidence suggests that repressive chromatin can spread from the viral LTR onto adjacent chromatin [21–23] (Fig. 2d). Silencing of LTR-directed transcription eventually causes loss of the viral transactivator protein TAT, which is of significant consequence because this stabilizes the provirus in a latent state by preventing viral mRNA elongation by RNA polymerase from the core promoter [19, 24]. A variety of additional mechanisms contribute to, and reinforce, maintenance of the latent provirus by preventing the expression of viral transcripts, including the effects of cellular miRNAs [25], and anti-sense transcription from the 3′ LTR of integrated HIV-1 which contributes to recruitment of repressive chromatin complexes [26].

There appear to be several pathways for the production of latent provirus. HIV predominately integrates within transcribed genes, due to interaction of the viral integrase with mRNA processing factors [27, 28]. However, integration at some specific chromosomal locations causes a greater tendency to produce latency. For example, transcriptional interference produced by nearby promoters inhibits expression from the HIV-1 LTR [29]. Additionally, the use of dual-reporter HIV derivatives bearing internal constitutively expressed reporters has indicated that ~ 50% of newly infected cells harbor transcriptionally silenced provirus within 24 h post-infection [30–32]. Establishment of immediate latent infection was also observed in cells infected with unmodified HIV strains [33] and is influenced by levels of basal signaling from the T cell receptor [31] (Fig. 1c). Immediate latency is associated with the binding of YY1, indicating that the establishment of this mode of latency is regulated by specific factors bound to the LTR soon after HIV genome integration [34]. Interestingly, the mechanism(s) for establishment of immediate latency can apparently overcome positive regulation by Tat [21]. Additionally, and important to this review, recent observations indicate that the pathway for production of latency may influence the spectrum of agonists that promote HIV reactivation [35].

## The latent HIV reservoir in patients on ART

HIV-1 infects cells through binding of HIV gp120^env^ with the CD4 membrane glycoprotein, and therefore all cell types expressing this marker could potentially accommodate infection (Table 1). However, the spectrum of target cells is modified by interaction of gp41^env^ with CD4 in combination with the co-receptors CXCR4, CCR5 and to a lesser extent CCR3 [44]. These chemokine receptors are expressed on the surface of T cells, macrophages, eosinophils and microglial cells (Table 1). Hematopoietic stem cells from human fetal liver also express CD4, but expression of the co-receptors appears to be heterogeneous, particularly for CXCR4 [45]. Human hematopoietic stem cells can be infected by HIV in vitro [18, 22, 32] and these cells may represent an important reservoir of virus in a subset of patients on ART [46]. The success of therapeutic strategies towards a cure seemingly will be strongly influenced by the spectrum of cells infected with latent provirus. Importantly, for example, the ability of HIV to infect the stem cell population may limit therapies involving specific depletion of only differentiated CD4^+^ populations, and overall, infected stem cells might represent an insurmountable obstacle towards a cure for some individuals, because these could presumably regenerate an infected T cell population upon differentiation. Additional cell types that lack CD4 receptors, including epithelial cells and astrocytes (Table 1) can become infected by syncytial fusion with infected CD4^+^ cells, but the significance of these infections for long term stability of the viral reservoir in patients on ART has not been determined [14, 47].

An important consideration relating to target cell specificity involves the half-life of latently infected cells in vivo. Most attention has been focused on CD4^+^ memory T helper cells, which have a lifespan of up to several years (Table 1), and are presumed to represent a primary reservoir of virus in patients on ART [48]. Studies indicate that the resting T helper cell population in HIV patients on ART may harbor ~ 1 replication competent viral genome per 10^6^ CD4^+^ cells [49]. This implies that, with a peripheral T cell population estimated at ~ 10^12^, patients on ART may harbor roughly 10^6^ latently infected cells. Interestingly, recent analysis indicates that the majority of HIV infected cells are derived from the proliferation of a limited number of clones in patients after 1 year on ART [50]. Considering that the estimated half-life of latently infected memory cells is about 4 years (Table 1), it has been predicted ~ 88 years of treatment would be required for this population to decay to a level that would be equivalent to a cure using current ART regimens [51]. These staggering estimates in part have contributed to the current push towards development of strategies to eliminate infected cells from patients, as current therapies are likely to become untenable over the long term with an aging patient population where side effects of treatment become evident, in addition to the inevitable occurrence of resistant HIV variants [10].

Infection of monocyte-derived macrophages and microglial cells (Table 1) also plays a significant role in dissemination and maintenance of HIV in patients on ART. Accordingly, mature macrophages appear to serve as a primary reservoir of HIV-1 in the humanized BLT mouse model [52]. Monocytes migrate to a variety of tissue compartments where they differentiate into macrophages and dendritic cells and also cross the blood–brain barrier as differentiated microglial cells. Although the life span of these cells may be shorter than T memory cells (Table 1), they can contribute to long-term persistence by their capacity to infiltrate a variety of tissue compartments where concentrations of antiretroviral drugs may not be sufficient to completely suppress HIV replication, known as sanctuary sites [53], and where the infection can be disseminated through low level/stochastic viral replication [54, 55]. For example, and particularly important, infiltration of latently infected microglial cells into the central nervous system may establish an isolated viral population that is impenetrable by current ART and may act both as a reservoir for viral maintenance, as well as contributing to neurological disorders in the ageing HIV-infected population [56]. Additionally, infected monocytes and macrophages are relatively resistant to the cytopathic effects of HIV replication and consequently, despite their shorter life span these cells may slowly release virus, which may contribute to viral persistence in patients over a long period [47].

## Prospects for a cure for HIV/AIDS

**Fig. 3:**
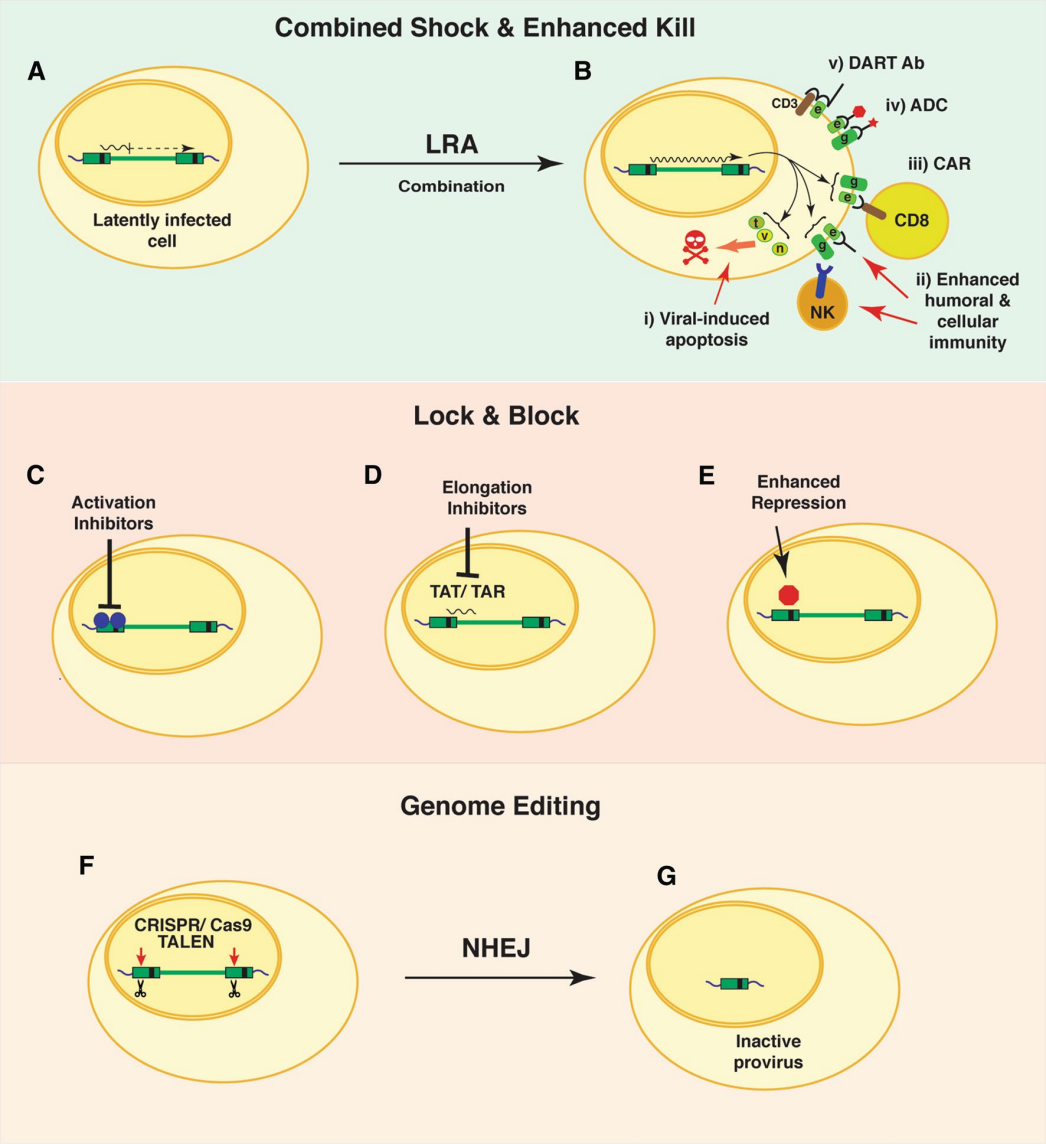
Potential strategies to eliminate cells latently infected with HIV-1. Combined Shock and enhanced Kill. Cells latently infected with HIV-1 provirus may produce sporadic transcripts (**A**), which mostly terminate shortly upon initiation because of RNA Polymerase II pausing factors (DSIF, NELF). Occasional transcripts may produce viral gene products, including the viral transactivator TAT. Combinations of latency-reversing agents (LRA) may be employed to produce broad and robust reactivation of provirus mRNA expression and production of viral gene products (**B**) (g, gag; e, env; t, tat; v, vif; n, nef). Killing of cells with reactivated provirus could be encouraged by additional intervention, including: (i) enhanced cellular apoptosis induced by viral proteins; (ii) enhanced cellular and/or humoral immune responses towards HIV-infected cells; (iii) production of designer chimeric antigen receptor (CAR)-expressing CD8 T cells that target HIV *env* or *gag* gene products expressed on the surface of infected cells; (iv) antibody drug conjugates (ADC) using *env*- or *gag*-specific antibodies coupled to toxic effectors; (v) recombinant Dual Affinity Retargeting Antibodies (DART) may promote specific elimination of specific subsets of HIV-1 infected cells following reactivation of provirus through recognition of a cell surface marker (CD3) in combination with viral gene products (*env*). Lock and block. Therapeutic intervention that prevents the expression of HIV gene products could “lock down” sporadic expression in latently infected cells, to prevent re-emergence of virus when anti-retroviral therapy is removed. Such strategies may involve inhibiting factors that activate transcription from the HIV LTR (**C**), prevent elongation of RNA transcripts from the viral LTR promoter (**D**) or enhancing effects of repressor proteins bound to the LTR regulatory region (**E**). Genome Editing of HIV provirus. Recombinant TALEN or CRISPR/Cas9 gene editing molecules delivered to latently infected cells designed to produce double-stranded cleavage at highly conserved regions of the provirus genome (**F**). Double-stranded breaks repaired by non-homologous end joining results in deletions of the HIV genome that prevents further replication (**G**)

Two infected individuals on ART have been effectively cured of HIV infection by a process involving immune depletive treatment for leukemia or lymphoma, followed by allogenic transplantation of hematopoietic stem cells from compatible donors expressing the naturally occurring CCR5Δ32 mutation, which prevents infection of CD4^+^ cells by HIV [57, 58]. These encouraging interventions demonstrate that a cure for HIV infection is possible if a sufficient fraction of latently infected CD4^+^ cells can be replaced with uninfected HIV-resistant stem cells. Unfortunately, this strategy is not feasible for the vast majority of infected individuals who are unlikely to be matched with compatible CCR5Δ32 donors. Consequently, the development of a routine effective cure will inevitably require the elimination of latently infected reservoirs, preventing transcription of the latent provirus, or destruction of HIV provirus by gene editing [59]. A variety of approaches based these general strategies have been proposed (Fig. 3).

A major limitation to the feasibility of most curative strategies for HIV/AIDS relates to identifying and targeting the latently infected population. Latently infected cells, as defined by complete repression of HIV transcription, theoretically would be devoid of viral proteins and therefore antigenically indistinguishable from non-infected cells. However, some evidence suggests that most latently infected cells produce sporadic occasional viral transcripts (Fig. 3A), through stochastic mechanisms that may maintain low levels of gene products that could produce a unique cellular identity, either directly, or indirectly by affecting the expression of host cell proteins [60]. Indeed, studies of HIV provirus in established cell lines indicates ongoing low-level replication in otherwise latently infected cells [21, 61]. Furthermore, infection by HIV, and most viruses, perturbs numerous signaling events resulting in massive changes in post-translational modifications of proteins [62]. Consequently, it is possible that cells with repressed integrated provirus may retain a molecular signature that is distinctive of the HIV infection which persists after viral gene products have disappeared. Whether markers produced by these potential mechanisms will be useful for the detection of the latently infected population in patients on ART remains to be determined.

Nevertheless, to enable specific targeting of the latent HIV infected population, there is considerable interest in identifying “biomarkers” of these cells that could serve as the focus for specific therapies. A useful biomarker would likely represent a cell surface molecule that could distinguish latently infected cells from their uninfected counterparts. One study reported that cells latently infected with HIV express significantly higher levels of CD2 than corresponding uninfected CD4^+^ T cells [63]. More recently, the low affinity FcγIIa immunoglobulin chain receptor, CD32a, was observed to be expressed in latently infected CD4^+^ T lymphocytes, but not uninfected T cells [64]. However, CD32a is only expressed in ~ 50% of latently infected T cells but is also expressed in cells of myeloid lineage. Additionally, recent analysis has disputed whether latently infected CD4^+^ cells specifically express this marker [65]. Altogether, because neither of these markers specifically delineate cells latently infected with HIV, their utility for the elimination of this population is questionable. Although efforts to identify biomarkers are ongoing, it seems likely that a combination of surface molecules may be required for therapeutic purposes to specifically and effectively target this population.

## Exposing and eliminating latently HIV-infected cells: Shock and Kill

Viral infections are normally cleared because the immune system recognizes pathogen proteins expressed on the surface of infected cells, which are eliminated by cell-mediated cytotoxicity [66]. In this respect, cells latently infected with HIV are essentially invisible to the immune system because they bear no virus-specific macromolecules that could distinguish them from uninfected cells. Consequently the “Shock and Kill” strategy for eliminating latently infected cells [67], and occasionally more recently termed “Kick and Kill” [68], is based on the premise that therapeutic intervention to force reactivation of HIV provirus [69], and expression of viral proteins would expose these infected cells to elimination by an immune response or virus-induced apoptosis [70] (Fig. 3B). Multiple parameters will influence the feasibility of this approach. HIV-1 transcription is regulated by cell signaling pathways linked to T cell receptor (TCR) engagement and T cell activation responses [17] (Fig. 2a). Therefore a major challenge is to force induction of viral expression without promoting global T cell activation, which can have life-threatening toxic effects by causing a “cytokine storm” otherwise known as cytokine release syndrome [71]. Additionally, the success of “Shock and Kill” will depend upon a sufficiently broad and robust induction of viral transcription, coupled with a corresponding competent immune response capable of purging the latently infected population [72]. Various reports of initial clinical trials [73] have revealed that both the “Shock” and the “Kill” parameters currently have limitations that will require further development [74], importantly considering that latently infected cells seem to be particularly resistant to killing by CD8^+^ cytotoxic T cells [75].

### Development of an effective “Shock” to expose latently infected cells

One significant obstacle towards an effective “Shock” relates to differential responsiveness of proviruses integrated at various chromosomal locations. HIV-1 integrates into the chromosome predominately within transcribed regions [76]. Several studies indicate that responsiveness of HIV provirus to T cell signaling and chromatin modifying agonists is strongly determined by the site of integration, and that regulation of viral transcription becomes influenced by the flanking chromatin environment [21]. Most integrated provirus is responsive to T cell signaling, which can be mimicked by treatment with phorbol 12-myristate 13-acetate (PMA), in combination with ionomycin, or cross-linking of the T cell receptor [77, 78]. However, in contrast, only a minor subset of provirus is induced, and only partially, by treatment with individual chromatin modifying agents, including HDAC, or histone methyltransferase (HMT) inhibitors [79]. Furthermore, in general, inhibitors that affect chromatin modifying enzymes mechanistically only produce an increase in basal transcription and thus cause a minor effect overall relative to induction that occurs as a consequence of T cell activation [79]. Additionally, signaling agonists on their own, including ionomycin, PMA, and its various analogs, as well as interleukin-2 (IL-2), interleukin-7 (IL-7), and Toll-like receptor 7 (TLR-7) agonists induce only a sub-population of provirus integrants, and in general induce transcription to a significantly lower level compared to T cell receptor stimulation [79]. Therefore, exposing a significant proportion of the latently infected population is not likely with a single agonist. Consequently, there is a recent trend towards the development of combinations of reagents that affect multiple pathways to produce broader and synergistic transcriptional responses [79, 80].

### Latency reversing agents (LRA) for shocking expression of HIV provirus

Regulation of HIV transcription is more complicated than for a typical signal-responsive host gene (Fig. 1). The latent HIV genome responds to multiple signaling pathways downstream of the T cell receptor (Fig. 2a), in addition to a variety of cytokines and innate immune stimuli [17, 81]. The enhancer of the HIV-1 LTR binds many transcriptional activator proteins (Fig. 1a), which mediate response to these signals, and overall, approximately 30 different factors have been found to interact with sequences within the 5′ LTR region [18]. Additionally, the viral transactivator TAT promotes transcriptional elongation from the core promoter (Fig. 1a), by recruitment of the pTEFb complex, phosphorylation of the RNA polymerase II C-terminal repeat domain, and inhibition of the pausing factors NELF and DSIF [82]. Consequently, considering the multitude of potential mechanistic opportunities for positive regulation, it might be expected that a large variety of chemical interventions would be capable of producing elevated expression from the LTR.

Initial studies to reactivate HIV provirus for the purpose of clearing the latently infected population involved the use of cytokines, including IL-2 and IL-7 [81] (Table 2). An early study showed that patients who received IL-2 in combination with ART had lowered counts of resting memory CD4^+^ T cells than patients who received anti-viral drugs alone [101], but this treatment was unable to eliminate latently infected cells. Additional signaling effectors used for latency reactivation in clinical trials have included agonists for TLR-2, TLR-7 and TLR-9 [102, 103] (Table 2).

**Table 2:** Classes of HIV-1 provirus latency reversing agents

	Examples	Txn. target(s)^a^	References
**Cytokines/receptor agonists**			
Interleukins	IL-2, IL-7, IL-15		[83]
TCR/Co-receptor activators	Maraviroc.		[84]
Toll-like receptor (TLR) agonists	TLR2, 3, 7, 8, 9 agonists		[85]
**Epigenetic modifiers**			
HDAC inhibitors	Vorinistat, panobinostat, AR-42, MS-275, chidamide	HDAC1, 2, 3	[86]
Histone methyltranserase inhibitors	Chaetocin, AZ505	Suv39H1, SMYD2	[87]
**Intracellular signaling modulators**			
PKC agonists	Ingenol EK-16A, gnidimacrin, bryostatin, SUW133, PEP005/Ingenol-3-angelate, Prostratin, Bryostatin-1	NFκB	[88]
AMPK activators	Dibutyryl-cAMP		[89] [90]
JAK/STAT agonists	Benzotriazole, benzazole	STAT3	[91]
IAP agonists	Debio1143	NFκB (non-canonical)	[92]
**Transcriptional elongation regulators**			
BET inhibitors	JQ1, MMQO (8-methoxy-6-32 methylquinolin-4-ol), UMB-136, RVX-208, PFI-1, OTX015	TAT/pTEFB	[93]
Cdk9 activators	Chalcone, Amt-87	pTEFB	[94]
**Unclassified**			
Anti-oxidant	Auranofin (AF)	NFκB	[95]
AKT modulators	Disulfiram, 57704		[96]
Sphingosine-1-phophate receptor 1 (S1P1) agonist	SEW2871	NFκB	[97]
Protein phosphatase 1	SMAPP1	pTEFb	[98, 99]
SMAC mimetics	SBI-0637142	NFκB	[100]

^a^HIV LTR-associated transcription factor stimulated by the LRA

The second class of “Shock” reagents are represented by inhibitors of epigenetic modifying enzymes (Table 2), namely histone deacetylases (HDAC), and histone methyltransferases (HMT); as mentioned above transcription of the HIV provirus is silenced by a combination of epigenetic mechanisms mediated by these enzymes [23] (Figs. 1b, 2d). Notably, many groups have demonstrated that chromatin modifying inhibitors are capable of inducing HIV provirus in a variety of cell line models, as well as cultured latently infected peripheral blood mononuclear cells (PBMCs). HDAC inhibitors, in particular had recently already been used in experimental trials for the treatment of various cancers, and consequently the earliest trials of shock and kill for patients on ART were performed with the HDAC inhibitors, valproic acid, vorinostat, and more recently panobinostat and romidepsin [81, 104]. Several of these studies were ongoing for at least 2 years, however, despite detectable induction of viral replication, in no case was a significant decrease in the overall latently infected population observed [68]. Although disappointing, in retrospect this outcome is not surprising given more recent results indicating that the site of chromosomal integration can affect HIV provirus response to various agents in vitro [21]. Furthermore, the fact that HDAC inhibitors were capable of inducing viral replication in vivo, but did not affect the latent reservoir in these trials indicates that the “Kill” parameter was not effective in clearing cells where HIV replication had been induced [70]. This suggests that patients on ART do not produce an adequate anti-HIV response, and also that few of these cells are eliminated by apoptotic mechanisms [105]. Importantly, these initial trials revealed limitations of both the shock and kill parameters, and ultimately have led to new efforts towards improving the strategy. Most important was realization that combinations of LRAs that target separate pathways for viral induction will likely be required to produce a broad and synergistic response to expose a significant portion of the latent population [70].

A third class of LRA includes compounds that modulate protein kinases in signaling pathways upstream of transcription factors that bind the LTR (Fig. 1a), and are normally regulated by cytokine signaling or T cell receptor engagement (Fig. 2a) (Table 2). This class includes the PKC agonists byrostatin, ingenol-3-angelate (PEP005), prostratin, and related molecules. Activation of PKC in T cells by these agonists cause induction of LTR transcription through activation of NFκB but produce a weaker response than molecules such as PMA [88]. Several of these compounds have been used in clinical trials [106]. NFκB can also be activated by a non-canonical pathway downstream of TNF receptors through NFκB-inducing kinase (NIK). An inhibitor of apoptosis agonist (IAPa, Debio1143) was recently shown to activate this pathway by promoting degradation of the non-canonical NFκB pathway inhibitor BIRC2 [92]. Dibutyrl-cAMP also acts as an HIV latency reversing agent through stimulation of protein kinase A signaling in several cell line models, alone and in conjunction with HDAC inhibitors [89, 90].

Productive transcription from the HIV-1 5′ LTR requires the expression of viral TAT protein, which binds the nascent TAT-response element (TAR) of the 5′ mRNA stem loop associated with paused RNA PolII complexes [107] (Fig. 1a). Binding of TAT to TAR recruits the super elongation complexes, represented by pTEFb and ELL2 [108]. Phosphorylation of the negative regulatory factors NELF and DSIF by Cdk9 of pTEFb relieves their inhibitory effect on transcriptional elongation, while phosphorylation of serine 2 of the RNA PolII CTD heptapeptide repeat promotes escape from the pre-initiation complex at the core promoter [109]. ELL2 functions to suppress transient pausing of RNA Pol II [110]. On the HIV-1 LTR the BET (bromodomain and extra terminal domain) protein, Brd4 interacts with pTEFb and inhibits binding of TAT, thereby inhibiting transcriptional elongation [110]. A variety of compounds known as BET inhibitors, typified by JQ1, cause reactivation of HIV transcription by antagonizing the negative effect of Brd4 on TAT [111] (Table 2). JQ1 also promotes release of pTEFb from the 7SK snRNP, making it available for recruitment to the LTR by TAT [112]. Because the BET inhibitors promote TAT-induced HIV transcription, it wouldn’t be expected that these compounds should induce latent HIV transcription that had been silenced for significant lengths of time, presumably where TAT protein is not expressed. Accordingly, BET inhibitors typically produce a delayed response of LTR-directed transcription, which is thought to be initially dependent upon stochastic basal expression [113]. Importantly, however, BET inhibitors produce a strong synergistic response with a variety of other latency-reversing agents [79]. In addition to its interaction with pTEFB, Brd4 also binds a variety of additional transcription and replication factors, and therefore it is possible that their effect on HIV LTR transcription may be modulated through other factors as well [114].

The final class of latency-reversing agents are referred to as “Unclassified” (Table 2) compounds since they typically were identified as previously utilized drugs, that were found to induce HIV provirus expression in vitro, but their precise mechanism of action towards this effect has not yet been precisely elucidated. For instance, disulfiram is an acetaldehyde dehydrogenase inhibitor and anti-alcoholism drug, which induces HIV-1 transcription in vitro [115], and is currently in clinical trials in combination with additional LRAs in infected patients on ART [104]. Disulfiram causes depletion of the phosphatase tension homolog, PTEN, which activates Akt signaling [91]. Although the precise mechanism for induction of HIV transcription by this pathway has not been determined, an additional Akt agonist (57,704) identified in a small molecule screen was also found reactivate virus expression in cell line models [96]. Similarly, auranofin, an organogold compound previously employed as an anti-inflammatory/anti-rheumatic agent, induces heme oxygenase expression and decreases anti-oxidant response in T lymphocytes, which induces HIV provirus expression [95]. These effects correspond with activation of p38 MAPK, mitochondrial depolarization and release of peroxides, which may cause induction of HIV through activation of NFκB in T memory cells. Serendipitously, it was also that found that SMAC (second mitochondria-derived activator of caspases) mimetics are capable of reversing HIV latency by an undefined mechanism. SMAC mimetics are small molecule compounds which antagonize inhibitors of XIAP, cIAP1, and cIAP2, and are currently in clinical trials for the treatment of solid tumors [116]. Interestingly, the SMAC mimetic SBI-0637142 produces synergistic induction of HIV expression in combination with HDAC inhibitors and causes apoptosis of latently infected CD4^+^ T cells in which replication has been reactivated [100]. Numerous additional small molecules that induce HIV expression in vitro have been identified from compound screens, but for most, mechanisms have not been determined [79]. Identification of additional novel agents that reverse HIV latency is expected to reveal further details of HIV provirus transcriptional regulation, which should lead to additional options for effective shock therapies. From initial clinical trials it has become apparent that a single small molecule “shock” effector is likely not sufficient to expose a significant proportion of the latent population [117]. Thus, the focus for further development is typically directed at identifying combinations of latency-reversing agents that synergistically induce broad and robust transcriptional responses [79, 118].

### Recombinant biomolecules for reversing HIV latency

LRA cytokines and compounds (Table 2) may also be used in combination with recombinant macromolecules for reversing HIV latency. For example, recombinant HIV TAT delivered in exosomes causes strong activation of provirus expression when added to latently infected cells [119], and an attenuated TAT protein (Tat-R5M4) produces synergistic activation of provirus in combination with HDAC inhibitors [120]. Similarly, CRISPR/nuclease deficient Cas9 (dCas9) and designer zinc finger proteins have been developed to force induction of HIV provirus, where the general strategy is to direct a transcriptional activation domain fusion to highly conserved elements on the 5′ LTR to cause constitutive expression. The CRISPR/dCas9 system involves the expression of dCas9 fused to a strong transactivation domain in combination with a guide RNA that recognizes a conserved element on the HIV LTR [121]. Similarly, transcriptional activator-like effector (TALE) zinc finger proteins, directed to conserved elements on the LTR and fused to a strong transactivation domain, also cause induction of latent HIV provirus [122]. The potential advantage of these strategies is that recombinant constitutive activator fusions are likely not subject to chromosomal position effects or cell signaling pathways, and possibly could induce a larger proportion of the latent provirus population. Furthermore, studies in both yeast and mammalian cells have shown that a single potent transcriptional activation domain, such as that encoded by HSV-1 VP16 [123], can overcome repressive effects caused by epigenetic silencing [124]. Consequently, these strategies could provide an effective shock to the latent population, pending development of delivery and expression systems towards appropriate target cells. An additional approach related to these possibilities may involve delivery or expression of molecules that inhibit the function of micro RNAs (miRNA) that suppress translation of HIV-1 encoded proteins. For example, complementary antisense RNAs were shown to block the inhibitory effect of miRNAs in latently infected T cells to promote viral production in resting T cells [125].

### Improving the “kill” of cells expressing reactivated provirus

While a variety of strategies are capable of forcing induction of provirus in latently infected cells, initial clinical trials with the shock and kill approach have shown that the immune system in ART patients is unable to produce a sufficient anti-HIV cytotoxic CD8^+^ T cell response to eliminate a significant proportion of cells in which replication has been reactivated [70]. Consequently, successful shock and kill will also require the development of complementary interventions that improve capacity to eliminate cells expressing viral antigens by enhancing cell-mediated immunity, or by facilitating apoptosis of HIV-infected cells (Fig. 3B). To this end, several strategies to improve HIV-1 specific cellular immune responses may prove effective when applied in combination with the LRAs discussed above.

Early after infection, the HIV accessory proteins TAT, Nef and Vpr interfere with cellular apoptosis mechanisms to encourage viral replication, but later Env and Vpu in combination with TAT, Nef and Vpr promote apoptosis by a variety of mechanisms [70]. Accordingly, it may be possible to enhance killing of cells harboring reactivated HIV provirus by inhibiting anti-apoptotic activity of these viral proteins and/or driving their pro-apoptotic functions [126] (Fig. 3B i). Accordingly, one report described the development of an artificial phosphoinositide, designated L-HIPPO which binds HIV-1 Pr55^gag^ and prevents translocation of virus to the plasma membrane thereby inhibiting virus release by budding. These “locked-in” virus particles were shown to cause apoptosis of the host cell [127]. Direct modulation of cellular apoptotic regulatory mechanisms can also be used to encourage the killing of infected cells. Bcl-2 was shown to have an anti-apoptotic effect on HIV infected cells through binding Casp8, which inhibits cleavage by HIV protease. A Bcl-2 antagonist, venetoclax prevents this binding and instead allows interaction with Bak to initiate apoptosis of infected cells [128]. Similarly, proteomic analysis of latently infected cells treated with LRA revealed that p38/JNK stress kinase pathways were altered, and furthermore that treatment with the JNK inhibitor anisomycin caused enhanced cell death following HIV provirus reactivation [129]. Additionally, a subclass of protein kinase C agonists were shown to promote apoptosis of HIV-1 infected cells through caspase 3 [130]. Recombinant macromolecules may also be employed to promote the killing of cells where viral gene products have been reactivated. For example, an inactive ricin A fusion protein which becomes activated upon cleavage by HIV-1 protease, delivered to cells in nanocapsules, was shown to sensitize cells to killing upon treatment with LRA [131].

## Preventing HIV replication from the latent population, Lock and Block, or destroy

Diametrically opposing strategies from shock and kill include those that would involve disabling the capability of the provirus to re-emerge from latency altogether; such strategies are often referred to as “Lock & Block” (Fig. 3C, D). This could mean intervention to prevent the expression of viral RNA, even in cells that have been stimulated by activation signals. With this strategy, it may be possible to remove patients from the current ART and specifically target the latently infected population to “lock down” or prevent viral re-emergence and spreading of the infection to other cells [132]. Alternatively, a more aggressive approach would involve genome editing to impair the capacity of HIV provirus to generate further productive infection or to delete provirus genomes altogether [133] (Fig. 3F, G).

### Locking down HIV provirus replication

Small molecule compounds may be developed specifically to either block HIV reactivation or to enhance silencing and repression of the HIV provirus. For example, several compounds developed for this purpose inhibit the basal or signal-induced activity of NFκB [134]. The variety of LRA that can induce HIV expression (Table 2) reflects the fact that the LTR enhancer has binding sites for numerous transcription factors (Fig. 1a), which represents an advantage towards the shock aspect of shock and kill strategies. On the other hand, sensitivity of the HIV LTR to this variety of factors presents a challenge towards locking down expression by inhibition of a single activator, such as NFκB (Fig. 3C), which is not likely to prevent reactivation of the entire latent population. Consequently, a general strategy to “lock down” HIV provirus expression might be more effective by encouraging transcriptional repression, through recruitment of HDACs, histone methyltransferases, DNA methyltransferases, or polycomb repressive complexes. In unstimulated cells, these complexes are recruited to the HIV LTR by sequence-specific binding factors, including but not limited to CTIP2, SP1, NFκB p50, and TFII-I [18] (Fig. 1b). Possibilities for this strategy may involve the identification of molecules that either stimulate the function of these sequence-specific binding factors or antagonize their negative regulators (Fig. 3E). There are not currently any small molecule compounds or drugs with these properties, and consequently, this represents an important focus for future investigation.

Several reports have described possible intervention to cause constitutive repression of HIV-LTR transcription using recombinant fusion proteins and/or RNAs (Fig. 3E). For example, recombinant zinc finger DNA binding fusions that recognize highly conserved regions on the LTR (Fig. 1), fused to KRAB transcriptional repression domains, have been shown to repress transcription from the LTR and inhibit activation of LTR-directed transcription by T cell signaling [135]. A similar strategy was employed using the CRISPR/Cas9 system, where dCas9 fused to a transcriptional repression domain was co-expressed with a guide RNA recognizing conserved elements on the LTR [136]. Several related strategies were designed to impair HIV transcription by interference with the TAT/TAR interaction (Fig. 1a), interfere with TAT transactivation function [137], disruption of the TAR RNA structure or folding [138], or to act as TAR RNA decoys to sequester TAT-pTEFb in complexes that are incapable of activating HIV transcription [139] (Fig. 3D). Transcriptional silencing of human genes can in some instances be caused using small interfering RNAs (siRNA) that target promoter regions. This was shown to be the case for the HIV-1 LTR using 21 nucleotide double-stranded siRNAs that target the enhancer region (Fig. 1a). Silencing established with this strategy was associated with accumulation of repressive chromatin marks on the LTR and could be reversed with HDAC inhibitors [140].

Strategies involving recombinant proteins or RNAs will require a means to specifically target and deliver expression constructs or recombinant macromolecules to latently infected cells, and consequently, their success may depend upon identification of appropriate biomarkers. Nevertheless, implementation of one or more strategies described in this section may allow HIV-infected individuals to substitute ART with a “Lock Down” therapy, which may be advantageous in cases where patients have developed intolerance to the ART regimen, or where the virus has become resistant to current drugs [141]. Furthermore, because “lock down” strategies seek to suppress re-emergence of HIV from latency, this type of therapy would reveal if viral replication actually occurs at low levels in patients on the current ART. The expectation would be that locking down low levels of replication in latently infected cells would prevent viral spread, in which case it may be possible to completely cease therapy after a sufficient length of time to allow natural depletion of the latently infected population.

### Destruction of HIV provirus genomes

The potential application of genome editing strategies provides novel opportunities to treat previously incurable diseases, and this is also true for HIV infection. CRISPR/Cas9 and TALEN-based gene editing strategies have both been applied to delete HIV provirus genomes in cell culture [136, 142]. These potential strategies have focused on highly conserved sequences within the LTR [143]; presumably Cas9 or TALEN-mediated endonuclease digestion within the 5′ and 3′ LTRs would promote deletion of the virus genome through repair by non-homologous end joining (NHEJ, Fig. 3F, G). CRISPR/Cas9 was shown to effectively delete HIV-1 provirus genomes in cell culture in vitro, and in vivo using mouse and rat model systems [144]. As with all gene editing strategies, for therapeutic purposes, to progress toward clinical trials, the technology must be optimized to minimize off-target effects. Importantly, because uninfected human cells do not possess HIV sequences it should be possible to develop highly specific editing reagents. Additionally, delivery vehicles must be developed, such as viral vectors, including lentivirus or adeno-associated virus (AAV), or non-viral vehicles, including ribonucleoprotein complexes or lipid-based nanoparticles. Another consideration relates to specificity; to minimize side effects, limiting the delivery of gene editing reagents to the latently infected population would be preferable. Genome editing has also been proposed for HIV therapies that are directed at limiting spread of virus in infected patients, for example by disruption of the co-receptor target genes in susceptible cells, which would limit the opportunity for virus spreading in patients on anti-retroviral therapy [133, 145, 146].

## Immunotherapy of the latent HIV-infected population

In conjunction with the strategies described above, a variety of immune-based therapies to facilitate the elimination of HIV-infected cells are possible. Most proposed strategies of this type are directed at enhancing killing or clearance of infected cells, in which viral replication has been induced with LRA, or stimulating natural immunity towards HIV-infected cells [147] (Fig. 3A, B). Ideally, immunotherapeutic intervention should enhance both the humoral and cell-mediated responses against HIV infected cells. This may involve a combination of boosting an already existing innate and adaptive immune response, inducing a response to novel HIV immunogens, and/or by passive immunization or boosting immune responses by co-treatment with modulatory cytokines and interleukins [148]. In this respect, IL-15 treatment was shown to induce natural killer (NK) activity, which enhanced the elimination of cells treated with LRA [149] (Fig. 3B ii). Similarly, the protein kinase PLK1 was shown to inhibit anti-HIV response of dendritic cells, and PLK1 inhibitors may, therefore, be useful to encourage endogenous HIV immunity patients treated with LRA [150].

One example of therapeutic vaccination currently under examination involves Vacc-4 × [151], which is comprised of four peptides representing highly conserved regions of HIV p24 viral core protein. Moreover, several recombinant humanized antibodies are currently in use for various therapeutic purposes, or under evaluation in clinical trials [152]. A minority of HIV-1 infected individuals develop antibodies targeting vulnerable and conserved regions of the HIV-1 *Env* gene product; these are designated broadly neutralizing antibodies (bnAB) and are capable of neutralizing infectivity of a wide range of HIV strains. These bnAB may be useful for a variety of strategies, including passive immunization, or in combination with other strategies to promote clearance of infected cells. For example, bnAB may be useful for recognition and clearance of cells where replication has been reactivated by LRA therapy, and viral protein synthesis has resumed [153]. Accordingly, recent observations from a trial in rhesus macaques, have shown that prolonged treatment with AAV vectors encoding bnAB can reduce viral load to undetectable levels in otherwise untreated animals, providing proof of concept for a functional cure [154].

Antibody therapies may also be used to encourage apoptosis of cells with reactivated provirus by targeting surface markers such as B7-H1 [155] and the negative co-signaling programmed-death 1 (PD-1) molecules [156]. Since these immunoregulatory molecules play a role in HIV-mediated T cell exhaustion [157], antibodies that block signaling from these cell surface molecules could potentially also restore function to a large fraction of the exhausted HIV-specific CD8 T cell population and render them strongly responsive to HIV infected cells [105]. Additionally, antibodies recognizing cytotoxic T-lymphocyte-associated protein 4 (CTLA-4) could be used to block immune checkpoint pathways in reactivated cells [158].

Another interesting possibility for treatment of the latent HIV population may involve bispecific or dual affinity retargeting (DART) antibodies that could encourage interaction of activated effector CD8^+^ T cells recognizing the CD3 receptor, in combination with HIV-specific *gag* or *env* antigens expressed on reactivated CD4^+^ T cells [75, 159] (Fig. 3B v). Additionally, conjugates of antibodies with cytotoxic compounds or drugs can be used to target and kill specific cell types recognized by the antibody. Such antibody drug conjugates (ADCs) are currently in trials for the treatment of various cancers and include conjugates of a wide range of previously employed anti-cancer drugs, such as doxorubicin, 5-fluorouracil, methotrexate, and many others [160]. Additional related strategies involve the use of conjugated or chelated short-lived alpha particle emitting isotopes [161]. Combined with LRA treatment, such ADCs employing antibodies that recognize HIV *gag* or *env* gene products, including the broadly neutralizing antibodies, when armed with their toxic cargo may provide an efficient killing response of the latently infected population (Fig. 3B iv).

Targeted cellular immune therapies directed against specific cancers are currently under investigation, with encouraging results [162]. Similarly, the cellular immune response towards cells expressing reactivated HIV provirus may be encouraged by isolation of CD8^+^ T cells from patients and modifying their specificity towards an anti-HIV CTL response in vitro, and then reintroduced to the patient following expansion [163]. For this purpose, various strategies have been developed to activate CD8^+^ T cells in vitro by treatment with heterocylic peptides, or co-culture with dendrocytes [164]. A similar approach, but more technologically engaging, would involve designer immune responses, where chimeric antigen receptors (CAR) are produced as a fusion of the CD_3_ chain signaling domain with the epitope single chain recognition motif identified from HIV-specific broadly neutralizing antibodies (Fig. 3B iii). This fusion could be expressed in autologous CD8^+^ T lymphocytes using lentivirus gene transfer, and the cells reinfused into patients treated with LRA [159, 165]. Similar strategies may involve transduction of CD8^+^ T cells with engineered T cell receptor genes with specificity redirected towards HIV antigens [74].

## Conclusion and perspectives

The chief obstacles to eradication of HIV infection are the lack of an effective vaccine, the failure of ART to clear HIV from infected patients, and the restoration of immune responses capable of suppressing HIV replication after cessation of treatment. During the past decade, there has been a major shift in focus of many HIV/AIDS research groups towards the development of potential cures for this disease. This focus has been directed at new technologies to quantify and identify the latently infected cells [166], and strategies to expose and eliminate this population from patients on anti-retroviral therapy. Despite some successes of global UN/AIDS programs in certain regions, the HIV/AIDS pandemic continues to grow at an alarming rate. Many of the proposed strategies described here rely on newly developed technologies, including genome editing, designer immune modulation and recombinant antibody therapy, as well as high throughput small molecule screens. Accordingly, research towards this goal has resulted in the identification of an entirely novel class of drugs known as latency-reversing agents (LRAs). Importantly, recent recognition that any one of these newly developed drugs and strategies are unlikely to be effective on their own, has led to a focus on devising therapies involving combinations of treatments that could identify and eliminate the latent HIV population. Considering the extensive variety of ingenious potential novel strategies presented here, we suggest that a cure may be within reach. However, it is important to recognize that broad implementation of successful new therapies developed that target latently infected reservoirs may be unavailable to the majority of infected individuals in impoverished and developing nations because of their reliance on high-tech strategies. Consequently, we submit that with the prospective of potential cures in sight, it is imperative that longer-term research goals be directed towards devising cost-effective means of implementing curative therapies on a global scale.

## Abbreviations

AAV: Adeno-associated virus
ADC: Antibody drug conjugates
AIDS: Acquired immunodeficiency syndrome
ART: Anti-retroviral therapy
BET: Bromodomain and extra terminal domain
bnAB: Broadly neutralizing antibodies
BLT: Bone marrow, liver, thymus humanized mice
CAR: Chimeric antigen receptor
CTIP2: COUP-TF-interacting protein 2
CTL: Cytotoxic T lymphocyte
CTLA-4: Cytotoxic T-lymphocyte-associated protein 4
CRISPR: Clustered regularly interspaced short palindromic repeats
CTD: C-terminal domain of RNA polymerase II
DART: Dual affinity retargeting antibodies
DNMT: DNA methyltransferase
DRB: 5,6-Dichloro-1-*beta*-d-ribofuranosylbenzimidazole
DSIF: DRB sensitivity inducing factor
ELL2: Elongation factor for RNA polymerase II
HDAC: Histone deacetylase
HIV: Human immunodeficiency virus
HMT: Histone methyltransferase
IL: Interleukin
HSPC: Hematopoietic stem and progenitor cell
KRAB: Kruppel associated box
LRA: Latency reversing agent
LTR: Long terminal repeat
NELF: Negative elongation factor
NHEJ: Non-homologous end joining
NIK: NFκB-inducing kinase
NK: Natural killer
PBMC: Peripheral blood mononuclear cells
PKC: Protein kinase C
PMA: Phorbol 12-myristate 13-acetate
PTEN: Phosphatase tension homolog
pTEFb: Promoter of transcriptional elongation factor b
RNA PolII: RNA polymerase II
siRNA: Small interfering RNA
SMAC: Second mitochondria-derived activator of caspases
TALE: Transcription factor-like effector
TAR: TAT-responsive element
TAT: Transactivator
TCR: T cell receptor
TLR: Toll-like receptor
TNF: Tumor necrosis factor
WHO: World Health Organization
XIAP: X-linked inhibitor of apoptosis
